# Transcriptome profile of the sinoatrial ring reveals conserved and novel genetic programs of the zebrafish pacemaker

**DOI:** 10.1186/s12864-021-08016-z

**Published:** 2021-10-02

**Authors:** Rashid Minhas, Henry Loeffler-Wirth, Yusra H. Siddiqui, Tomasz Obrębski, Shikha Vashisht, Karim Abu Nahia, Alexandra Paterek, Angelika Brzozowska, Lukasz Bugajski, Katarzyna Piwocka, Vladimir Korzh, Hans Binder, Cecilia Lanny Winata

**Affiliations:** 1 International Institute of Molecular and Cell Biology in Warsaw, Warsaw, Poland; 2 Randall Centre of Cell and Molecular Biophysics, Faculty of Life Sciences and Medicine, King’s College London, London, UK; 3 Interdisciplinary Centre for Bioinformatics, University of Leipzig, Leipzig, Germany; 4 School of Human Sciences, College of Science and Engineering, University of Derby, Derby, UK; 5 Nencki Institute of Experimental Biology, Laboratory of Cytometry, Warsaw, Poland

**Keywords:** Zebrafish, Cardiac conduction system, Pacemaker, Sinoatrial node, Sinoatrial ring, RNA-seq

## Abstract

**Background:**

Sinoatrial Node (SAN) is part of the cardiac conduction system, which controls the rhythmic contraction of the vertebrate heart. The SAN consists of a specialized pacemaker cell population that has the potential to generate electrical impulses. Although the SAN pacemaker has been extensively studied in mammalian and teleost models, including the zebrafish, their molecular nature remains inadequately comprehended.

**Results:**

To characterize the molecular profile of the zebrafish sinoatrial ring (SAR) and elucidate the mechanism of pacemaker function, we utilized the transgenic line sqet33mi59BEt to isolate cells of the SAR of developing zebrafish embryos and profiled their transcriptome. Our analyses identified novel candidate genes and well-known conserved signaling pathways involved in pacemaker development. We show that, compared to the rest of the heart, the zebrafish SAR overexpresses several mammalian SAN pacemaker signature genes, which include *hcn4* as well as those encoding calcium- and potassium-gated channels. Moreover, genes encoding components of the BMP and Wnt signaling pathways, as well as members of the Tbx family, which have previously been implicated in pacemaker development, were also overexpressed in the SAR. Among SAR-overexpressed genes, 24 had human homologues implicated in 104 different ClinVar phenotype entries related to various forms of congenital heart diseases, which suggest the relevance of our transcriptomics resource to studying human heart conditions. Finally, functional analyses of three SAR-overexpressed genes, *pard6a*, *prom2*, and *atp1a1a.2*, uncovered their novel role in heart development and physiology.

**Conclusion:**

Our results established conserved aspects between zebrafish and mammalian pacemaker function and revealed novel factors implicated in maintaining cardiac rhythm. The transcriptome data generated in this study represents a unique and valuable resource for the study of pacemaker function and associated heart diseases.

**Supplementary Information:**

The online version contains supplementary material available at 10.1186/s12864-021-08016-z.

## Background

The cardiac conduction system (CCS) is an essential component of the heart across vertebrates [1, 2]. It is responsible for initiating and coordinating the electrical signals that cause rhythmic and synchronized contractions of the atria and ventricles [3]. In higher vertebrates, this system comprised of the sinoatrial node (SAN) (the primary pacemaker site), atrioventricular node (AVN) (the secondary pacemaker site), and the “wiring” of the ventricles, namely the Purkinje fibres [1]. Electrical impulses are first generated in the SAN and are rapidly propagated through the heart muscle cells (cardiomyocytes, CMs) of the atrium, resulting in atrial contraction [4]. The electrical impulses then reach the slow conducting tissues of the secondary pacemaker at the AVN, causing a delay before being passed on to the fast conducting Purkinje fibres, which form a network throughout the ventricular myocardium [3, 5]. The primary pacemaker consists of a specialized group of CMs with distinctive morphological and electrophysiological properties [6].

Studies from mammalian and fish model systems have shown that distinct components of the CCS derive from the CMs, where they were set apart early from working CMs and driven to adopt distinct morphological and physiological characteristics to perform their specialized functions [7]. The SAN, which serves as the primary pacemaker site, develops within the sinus venosus, an area located at the site of blood entry at the meeting point between the right atrium and superior vena cava. In murine embryonic heart, *Tbx3*-expressing CMs in the early heart tube give rise to the SAN pacemaker cells, where Tbx3 activates the pacemaker genetic program and suppresses that of working CMs [7–9]. These cells also retain the expression of LIM-homeodomain transcription factor *Isl1*, which is lost in differentiated CMs [10–12]. The pacemaker gene program causes the SAN pacemaker cells to retain a low proliferation rate and develop slow conduction through the expression of low conductance gap junction proteins such as Cx30 [13, 14].

The CCS is evolutionarily conserved in the building plan of the heart, and it indicates that the cellular and molecular mechanisms that drive the formation of pacemaker tissues are almost similar among vertebrates [2]. The zebrafish two-chambered heart with a single atrium and a ventricle has remarkable similarities to the human heart in terms of basal heart rate, electrophysiological properties, and action potential shape and duration [15–17]. Several zebrafish mutants have been described which display phenotypes closely resembling that found in various forms of human arrhythmia [18–22]. It suggests the high conservation of molecular pathways regulating heart conduction. However, despite its promise as a model for human cardiac arrhythmia, the zebrafish has a poorly characterized CCS. Unlike that in mammals, zebrafish pacemaker cells are difficult to distinguish morphologically from CMs. Although the zebrafish homologs of the pacemaker genes *tbx2b* and *tbx3a* are expressed in tissues which correspond to the pacemaker in mammalian heart [2, 23], no detailed analyses have been made to confirm the identity of these expression domains. Identification of the pacemaker domain in the zebrafish has relied on the mutually exclusive expression of marker gene pairs to distinguish pacemaker cells from CMs, such as *isl1*, which revealed a ring structure in the myocardium at the junction of sinus venosus and atrium [23]. Visualization of calcium activation wave using the fluorescently-labelled calcium transgenic line has traced pacemaker activity to a small number of cells residing within the sinoatrial ring (SAR) which served as initiation site [24]. However, to date, no live markers of the pacemaker have been available, which would enable direct visualization of its morphology and structure or isolation for molecular characterization.

A screen of enhancer trap lines generated through transposon-mediated transgenesis [25] has generated a collection of transgenic lines expressing reporter genes in different tissues or subdomains of the heart [26, 27]. In the transgenic line sqet33mi59BEt, GFP is expressed in a ring structure at the venous pole of the heart [26], which is in agreement with the known position of the pacemaker region described previously [23]. To characterize the molecular building blocks of pacemaker, we isolated cells of the SAR and profiled their transcriptome. We identified well-known conserved signaling pathways, including Wnt and BMP, as well as novel candidate genes involved in pacemaker development. Our results show that the SAR transcriptome profile exhibits similarities to that of the mammalian SAN in terms of the expression of several key genes regulating pacemaking activity as well as its development.

## Results

### Transcriptome profile of the zebrafish SAR

**Fig. 1:**
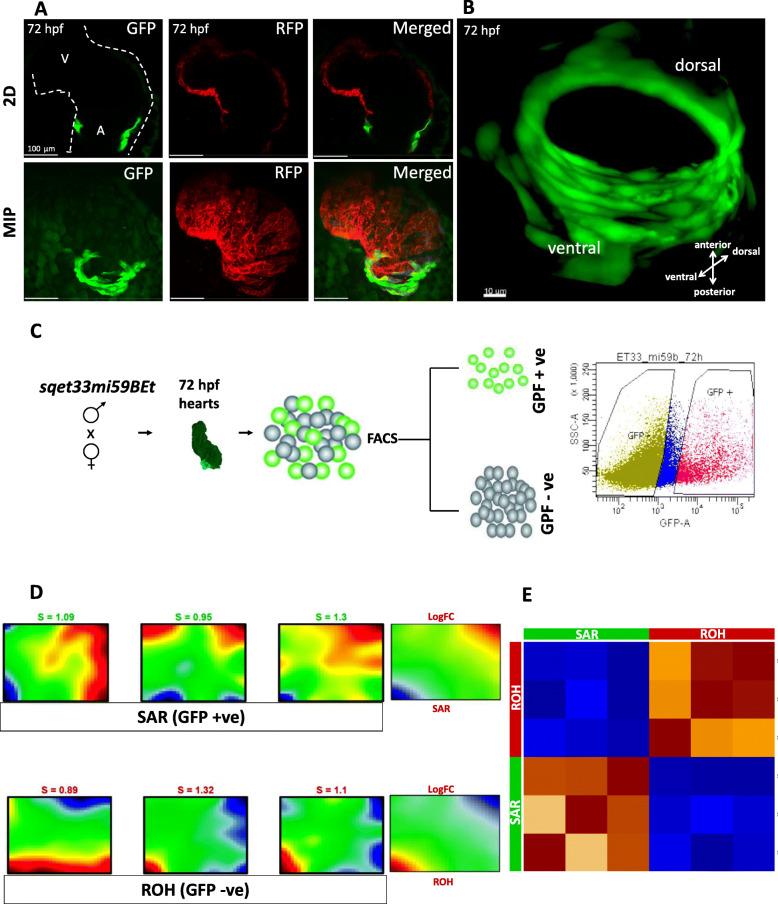
Morphological and transcriptional characterization of the zebrafish SAR. **A** In vivo confocal imaging of double transgenic sqet33mi59BEt x Tg (*myl7*:dsRed). **B** 3D reconstruction of the EGFP expression domain in the SAR at 72hpf. Individual channel signal threshold levels were adjusted for better visibility. **C** Schematic showing the strategy of in-cross of sqet33mi59BEt (preferably homozygous) to collect the GFP fluorescent embryonic hearts at 72 hpf followed by FACS sorting to GFP+ (SAR) and GFP- (rest of the heart). **D** Expression portraits of RNA-Seq data (dark red: strong overexpression, yellow and green colors: intermediate levels with low or no differential expression, dark blue: strong underexpression) showing the overexpression of metagenes in SAR (top panel) and the rest of the heart (bottom panel). The right-most portrait is the average of the three individual replicates of SAR and ROH, respectively. The indicated S-Value (silhouette value) is a measure of how similar an object is to its own cluster (cohesion) compared to other clusters (separation). **E** Pairwise Pearson’s correlation map between the replicates of SAR and the rest of the heart. 2D, 2-dimensional; MIP, maximum intensity projection

To localize the SAR and to distinguish it from the rest of the cardiomyocytes, the sqet33mi59BEt line was crossed with the transgenic line Tg (*myl7:*dsRed). In the double transgenic, dsRed-labeled CMs partially overlap with the small number of GFP-positive cells around the inflow region of the heart at 72hpf (Fig. 1A). To visualize the structure of the zebrafish SAR in more detail, we generated a 3D-reconstruction of the region expressing GFP in the sqet33mi59BEt transgenic line at 72 hpf (Fig. 1B) using light sheet fluorescence microscopy. At this stage, GFP-expressing cells make a compact ring of approximately 40 cells at the inflow region of the atrium, near the sinus venosus. This location corresponded to the reported location of the zebrafish SA pacemaker [23, 26, 28]. The SAR appears to contain six-seven layers of cells at the ventral side, while only two layers of cells were observed on the dorsal side.

To obtain GFP-expressing SAR cells, intact beating hearts were manually picked at 72 hpf under a fluorescent microscope, their cells subsequently dissociated and sorted using fluorescent activated cell sorting (FACS) (Fig. 1C). The average fraction of FACS-yielded GFP-positive events obtained represented between 5 and 13% of total singlet events (Figure S1A). To assure the cells were sorted correctly, the GFP transcript levels in the two sorted fractions were compared using qPCR. GFP expression was enriched in GFP+ as compared to the GFP− fraction (Figure S1B). In contrast, mRNA levels of *neurogenin1* (*ngn1*), a neuronal gene which serves as a negative control, was not detected in GFP+ and GFP− cells. Therefore, we concluded that the GFP+ fraction represents the SAR cell population, while the GFP- fraction represents rest of heart (ROH).

Transcriptomic data of 19,854 genes was analyzed based on Transcript Per Million (TPM) using the oposSOM R-package [29]. The expression portraits of SAR and ROH cell populations show two spots in opposite corners of the map, respectively, referring to opposing expression modules (Fig. 1D): in SAR, metagenes located in top-right corner of the map are overexpressed, while those in the bottom-left corner are underexpressed, and vice versa in GFP- samples, revealing antagonistic expression patterns between the two sets of samples. Pairwise correlation maps illustrating Pearson correlation coefficients were then calculated for all replicates of the SAR and ROH. All replicates of the SAR and ROH exhibit a strong negative correlation with respect to each other, underlining the antagonistic expression patterns as seen in the sample portraits (Fig. 1E).

### Genes and functional categories enriched in SAR cells

**Fig. 2:**
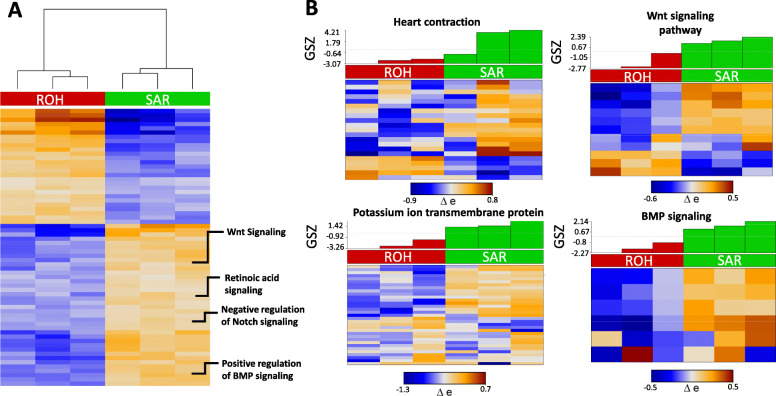
GO terms enriched among SAR overexpressed genes. **A** Functional annotation heatmap showing differentially expressed GO terms enriched in SAR (*p* < 0.01). **B** Heat map showing the expression pattern of genes related to heart contraction, potassium ion transport membrane protein, Wnt and BMP signalling in SAR

**Fig. 3:**
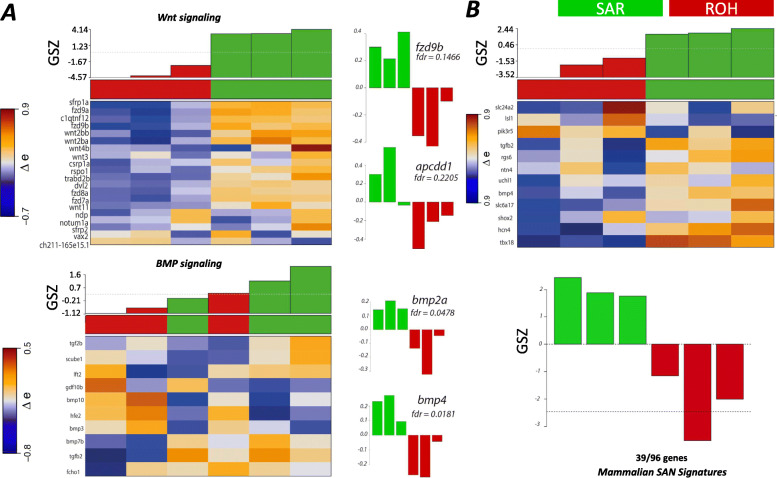
Molecular signatures and signalling pathways of SAR correlate with those in the mammalian SAN. **A** Heatmap showing the expression of components of Wnt/β-catenin and BMP signalling genes reported in [30] in SAN and ROH. Histograms of expression of the some of the key genes in both pathways are shown to indicate a gene-specific difference in the two sample sets. **B** Heatmap showing the expression of mammalian SAN genes reported in Vedantham et al. (2015) that have been compared with our SAR and ROH data set. A total of 39 out of 96 genes among the mammalian signature genes were overexpressed in our dataset

A total of 36 metagenes containing 730 genes were overexpressed in all 3 SAR replicates and underexpressed in ROH (Fig. 1D, top right red cluster, Table S1). In contrary, 24 metagenes and 562 genes were overexpressed in ROH and underexpressed in SAR (Fig. 1D, bottom-left red cluster, Table S1). To investigate molecular signatures specific to the SAR, we performed functional annotation analysis using oposSOM’s built-in gene-set library. Enriched GO terms among overexpressed genes in SAR cells include “cardiac muscle proliferation”, “heart contraction”, and “potassium ion transmembrane” (Fig. 2A, Table S2), which reflects the CM identity of these cells. In particular, the gene set under the “heart contraction” and “potassium ion transmembrane proteins” terms (Fig. 2B, Table S2) were overrepresented in SAR as compared to ROH. The gene encoding *wnt1*, a ligand of Wnt pathway, and *bmp4*, a ligand of BMP pathway, which have been reported to be expressed in the sinus venosus region [30], were among the top genes differentially expressed in SAR. Moreover, genes encoding transcription factors known to be responsible for pacemaker specification including *tbx5a* and *tbx18* were also overexpressed (Table S1) [31]. Unexpectedly, the pacemaker signature *isl1* was not among the differentially expressed genes in SAR compared to ROH. Although its transcripts are present in the SAR at comparable levels as the rest of the heart (Table S6), its expression was not detectable by whole mount in situ hybridization at 72 hpf (Figure S3). Studies have shown that the Wnt signalling promotes the specification of pacemaker cells in mammals and teleost [30, 32]. Our data set shows that genes related to the Wnt signalling were enriched in SAR as compared to ROH (Fig. 3A, top panel). We found that 55 genes of the Wnt signalling pathway were among the genes upregulated in SAR, such as *frizzled homolog 9b (fzd9b)*, *wnt2ba, wnt2bb,* and *wnt11.* Another signalling pathway crucial for pacemaker activity is the BMP signalling [3, 33]. Our data confirms that most of the components of this pathway, including *bmp2, bmp4* and *bmp7b* were overexpressed in SAR (Fig. 3A, lower panel).

SAN function is thought to be dependent on a 2-clock mechanism: the calcium clock and a voltage clock. The automaticity of the SAN is jointly regulated by the rhythmic spontaneous Ca2+ release from the sarcoplasmic reticulum and the cyclic activation and deactivation of membrane ion channels [34]. The hyperpolarization-activated, cyclic nucleotide-gated channel 4 (*hcn4*) subunit, which is known for its role in generating the pacemaker current [35], was one of the SAR’s 730 overexpressed genes. In addition, calcium channel subunit, T-type channels and potassium channel related genes (*kcnq1*, *kcnh2*) known to be overexpressed in pacemaker cells [36], were overexpressed in the SAR transcriptome (Table S3).

**Fig. 4:**
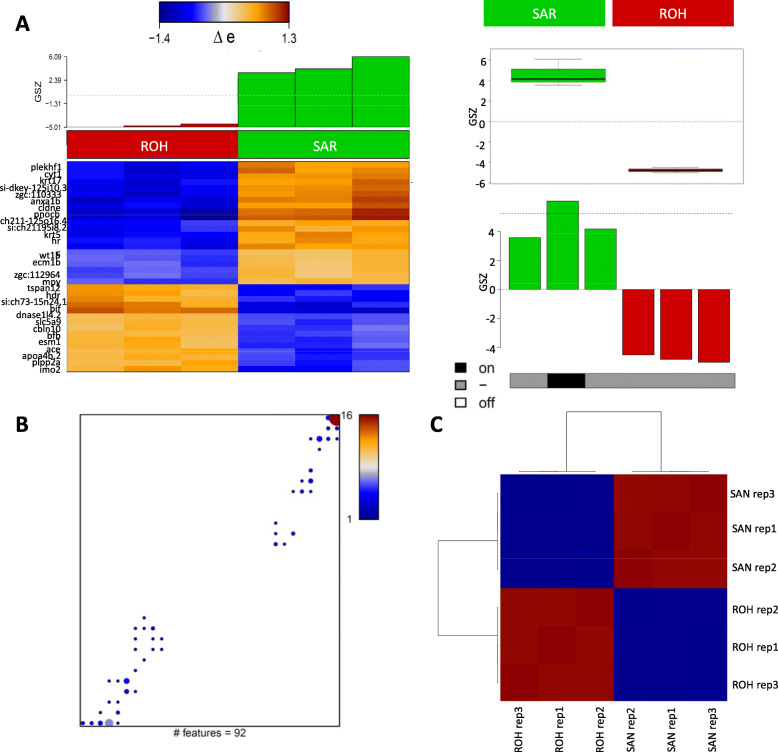
Genes overexpressed in SAR and ROH. **A** Genes differentially expressed between SAR and ROH were identified based on FDR (false discovery rate) < 0.1 comprising 92 genes (51 + / 46 -) with *p*-values < 0.0001. The boxplot and barplot show average expression of those genes in the samples and in the cell populations, respectively. The heatmap shows 92 genes deviating more than one standard deviation from average expression. **B** Mapping of differentially expressed genes into the SOM grid: Number of genes in the metagenes is color-coded (maroon> red> blue) with white areas representing metagenes not containing any of the 92 DE genes (FDR < 0.1). **C** Pairwise Pearson’s correlation map between the replicates of SAR and ROH based on the 92 differential genes only. Contrast between the two cell populations is enhanced as expected (compare to Fig. 1E). Red color represents 3 samples of sinoatrial ring while blue color indicates 3 sample of rest of the heart. SAR; Sinoatrial ring, ROH; rest of the heart

In order to identify novel pacemaker-specific genes, we performed differential expression analysis, showing the two main cell type-specific groups of transcripts. At the FDR (false discovery rate) significance level of < 0.1, we identified 92 genes (49 SAR-highly-enriched genes and 43 ROH-highly-enriched) (Table S4). Interestingly, expression of some genes previously not reported in pacemaker, such as *pard6a, prom2*, *wt1b*, *brd2a*, *atoh8*, *fn1b*, and *ldlrap1a,* were higher in SAR compared to ROH (Fig. 4A, Table S4). The gene maps show the distribution of these differentially expressed genes within the original SOM, one cluster forms at the top-right corner (overexpressed in SAR) and another at the bottom-left (overexpressed in ROH) (Fig. 4B) in agreement with the sample portraits shown in Fig. 1D. The difference between these two cell populations was also evident from the Pairwise Pearson correlation heatmap (Fig. 4C).

### Known mammalian SAN signatures are enriched in SAR

Several landmark studies have reported the transcriptome profile of mammalian SAN cells isolated by various methods. Vedantham et al. (2015) profiled the transcriptome of SAN cells isolated by laser capture microdissection from a sinus node reporter mouse line and identified differentially expressed genes between SAN and right atrium (RA) at different time points, yielding a set of SAN core genes. We found that 39 out of 96 mammalian SAN core genes identified in their study were among the top SAR-overexpressed genes. These include *tbx18, hcn4, bmp4, tgfb2,* and *slc6a17* (Fig. 3B; Table S5). More recently, mouse and human SAN molecular signatures were reported from cells isolated from Tbx3-driven reporter mouse and human fetal SAN cells (van Eif et al., 2019). We identified 26 out of 89 conserved SAN signature genes overexpressed in zebrafish SAR (Table S5; Figure S2). These include *cacnb1*, *camk4*, *bmp4*, and *hcn4.* Interestingly, when we compared our SAR-specific transcriptome with results of these two studies (39 and 26 genes), we found nine common genes: *shisa4, rgs6, bmp4, uchl1, sfrp5, hcn4, tub, boc,* and *chrnb4*. These nine genes might have a crucial role in SAN development and function (Table S5). Furthermore, several known calcium ion channels (*cacna1ab*, *slc8a4b*) [37], extracellular matrix genes (*mmp11a*, *thbs2b*) and neural crest genes (*foxd3,gria1a, smarca4a*, and *sox10*), which were reported to be expressed in SAN [33] were also overexpressed in our SAR dataset (Table S5).

**Fig. 5:**
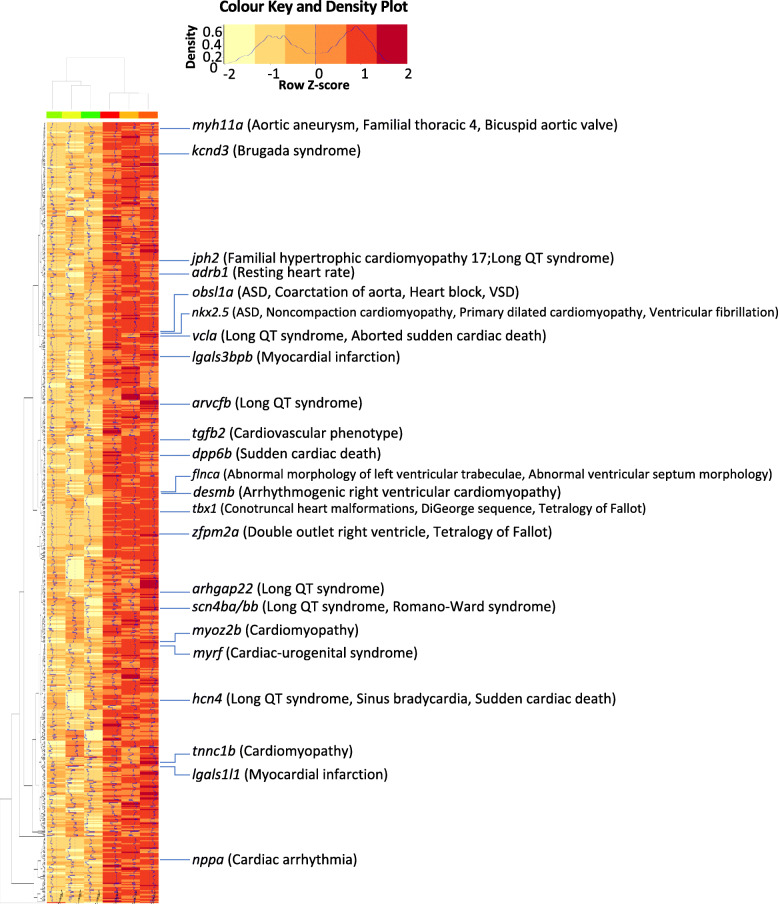
Orthologs of the SAR-overexpressed genes linked to human congenital heart diseases. Heatmap showing SAR-overexpressed genes whose human orthologues are associated with congenital heart diseases according to ClinVar phenotype entries

In order to assess the relevance of the zebrafish SAR transcriptome to human diseases, we ask whether any of the human orthologs of SAR-overexpressed genes were associated with congenital heart diseases. We found 24 genes implicated in 104 different ClinVar phenotype entries related to various forms of congenital heart diseases (Fig. 5, Table S7), among which are forms of cardiac arrhythmia, including Brugada syndrome, sinus bradycardia, sudden cardiac death, and Long QT syndrome.

### Knockdown of three SAR-overexpressed genes causes defects of heart morphology and physiology

To validate the relevance of SAR-overexpressed genes identified in our study to cardiac function, we selected three genes for functional analyses by MO knockdown. The candidate genes *pard6a, prom2,* and *atp1a1a.2* were not previously associated with cardiac function. *pard6a* encodes the adapter protein involved in asymmetrical cell division and cell polarization processes in the neural tube [38] . Prom2 localizes to basal epithelial cells and may be involved in the organization of plasma membrane microdomains [39]. It interacts with amigo1, the known interactor of the delayed rectifier voltage-dependent potassium channel KCNB1 [40, 41]. The Atp1a1a.2-related Atp2a1a.1 was associated with cardiocytes differentiation and cell migration during heart formation [42]. All three genes were expressed in the SAR (Figure S3) which supports their role in SAR function.

**Fig. 6:**
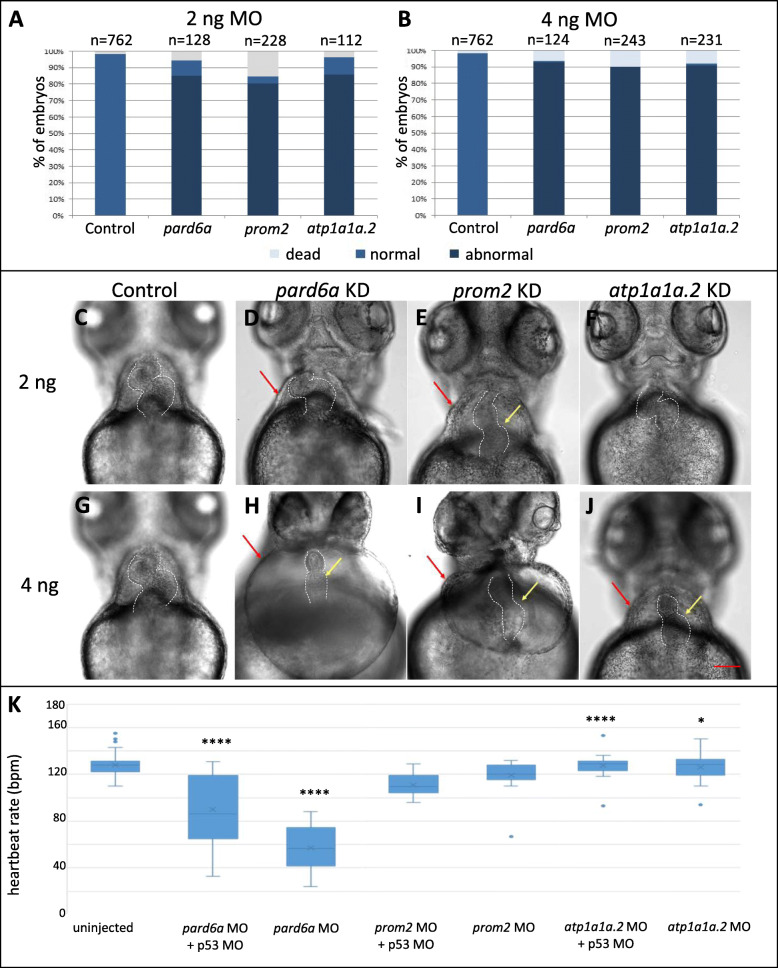
Morpholino mediated knockdown of three SAR-overexpressed candidate genes. **A**-**H** Heart morphology of control larvae and injected with 2 and 4 ng gene-specific morpholino against *pard6a, prom2*, and *atp1a1a.2* after 72 hpf. Red arrows indicate pericardial edema, yellow arrows indicate a failure in heart looping. **I**-**J** Percentage of morphological defects observed as a result of 2 and 4 ng of MO injections, showing dose-dependent effect. Scale bar: 50 μm. **K** Distribution of average heartbeat rate (in beats per minute, bpm) assayed in 72 hpf larvae as a result of knockdown with 2 ng or 4 ng of MO against *pard6a, prom2,* and *atp1a1a.2*. “*” indicates that the difference in heartbeat rate compared to that of control (uninjected) group was statistically significant. Table shows average heartbeat rate and Student’s T-tested *p*-value

Injections of 2 ng of gene-specific, translation blocking MO resulted in developmental delay in about 85% of larvae for *pard6a*, 80% for *prom2*, and 86% for *atp1a1a.2* (Fig. 6A). Injection of 4 ng of MO resulted in a high percentage of embryos with abnormal morphology: 92.7% for *pard6a*, 90% for *prom2*, and 90.9% for *atp1a1a.2* (Fig. 6B). Bright-field microscopy imaging revealed abnormalities in heart morphology. At 72 hpf, the heart in control larvae show normal cardiac looping with properly developed chambers and no cardiac edema (Fig. 6C, G). In individuals injected with 2 ng of *pard6a* MO, the heart morphology appeared unaffected apart from the presence of mild pericardial edema (Fig. 6D). In those injected with 2 ng of *prom2* MO, both chambers were present, but the hearts failed to loop and developed pericardial edema (Fig. 6E). Knockdown of *atp1a1a.2* injected with 2 ng of MO did not appear to affect heart morphology compared to the control group (Fig. 6F). Injection of 4 ng of *pard6a* or *prom2* MO resulted in a more pronounced pericardial edema, together with linearized heart tube (Fig. 6H, I). On the other hand, 4 ng of *atp1a1a.2* MO had a comparatively milder phenotype: both heart chambers were distinguishable although heart looping was not complete, accompanied by mild pericardial edema (Fig. 6J). This could be due to the redundant function of the related *atp1a1a.1*.

To test if MO-induced loss-of-function affects heart physiology, heartbeat rate in MO-injected embryos were measured and compared to that of uninjected control. The average heartbeat rate for uninjected larvae at 72 hpf, was 128 beats per minute (bpm) (Fig. 6K, *n* = 61), which was within the reported range (Poon and Brand, 2013). Average heart rate of embryos injected with 2 ng of *pard6a* MO was 102.25 bpm (*n* = 20; *p* = 5.77E-04), while those injected with 4 ng of MO had a heartbeat rate of only 57 bpm (*n* = 20; *p* = 1.59E-13), which indicated severe bradycardia (Fig. 6K). Injection of *prom2* MO did not result in significant changes in average heart rate (n = 20). In embryos injected with 2 ng and 4 ng of *atp1a1a.2* MO, average heart rate was 111 bpm (*n* = 21; *p* = 1.26E-10) and 119 bpm (n = 20; *p* = 1.28E-02), suggesting bradycardia (Fig. 6K).

Taken together, MO knockdown of each of the three candidate genes resulted in various forms of disruption to the heart morphology and heartbeat rate. All observed phenotypes were retained when p53 MO were co-injected with the gene-targeting MOs (Fig. 6K, S4), confirming the lack of off-target effects due to p53-dependent cell death [43]. Moreover, co-injection of 4 ng of MO for each candidate genes with varying levels of mRNAs encoding them (*pard6a* – 10 pg, *prom2* – 50 pg, *atp1a1a.2* – 5 pg) could rescue the observed morphological defects with varying degrees, providing further confirmation of the MO specificity (Figure S4).

## Discussion

The understanding of molecular mechanism underlying CCS development and function is one of the key challenges, which require addressing in order to develop better diagnosis and treatment for diseases related to pacemaker dysfunction. Several studies have reported the transcriptome profile of the SAN tissue isolated using laser capture microdissection in mouse [12] and TOMO-seq in zebrafish [30]. More recently, analysis of specific cell population of the mouse SAN was enabled by FACS to isolate specific populations of fluorescently-labelled cells from Tbx3+/Venus knock-in mice [33]. Their study revealed the SAN-enriched gene expression program, among which TBX3, SHOX2, ISL1, HOX family, BMP and NOTCH signalling, were conserved between human and mouse.

In this study we utilized a transgenic zebrafish line expressing GFP at the SAR region located between the sinus venosus and the atrium [26, 27], from which SAR cells were isolated using FACS. The location of GFP-positive cells in embryonic zebrafish heart and the overexpression of molecular markers *shox2* and *hcn4* in our data support the idea that pacemaker cells are a part of the GFP-expressing SAR and comprise cells involved in the origin of electrical current and contraction of the zebrafish heart [26] (Figure S2). Unlike the mammalian pacemaker signature, we observed that CM markers such as *nppa* was overexpressed while the gene encoding the slow conducting connexin Cx32.3 was underexpressed in the SAR transcriptome (Table S1). This suggests that the GFP-expressing region of the sqet33mi59BEt likely consists of a heterogenous population of cells, comprising of several cells with a definite pacemaker activity plus other cells, some of which may represent the pacemaker progenitors. This is in line with the previous observation by Chi and colleagues (2008) where the origin of activation could be traced to a small number of cells in this area.

We performed transcriptome data portraying using a self-organizing map that includes the expression patterns of all genes expressed in the SAR of 72 hpf zebrafish. Analysis of the SAR (GFP-positive cells) and ROH (GFP-negative cells) transcriptome highlighted two sets of genes: (a) genes overexpressed in SAR and (b) gene cluster overexpressed in ROH (Fig. 1). These genes are inversely expressed in both populations of cells. The 730 genes overexpressed in the SAR encompass many uncharacterized ones, indicating the potential significance of this dataset for revealing new genes and pathways involved in zebrafish CCS development and function. Interestingly, most of the SAR-highly-enriched genes have not been previously implicated in pacemaker development or function. Comparison of our dataset with recently published mouse SAN signatures [12, 33] highlights that many of the important SAN pacemaker genes are overexpressed in zebrafish SAR. However, a few known CCS markers including *isl1* and *shox2* were unexpectedly underrepresented in SAR as compared to ROH. It is possible that the expression of these genes, which play a developmental role, may not be as strong in the SAR by 72 hpf as at earlier stages. In addition, the heterogeneity of the SAR region, in which very few cells possess definite pacemaker activity, as well as their possible expression in other parts of the heart, may also contribute to their lack of overexpression compared to the rest of the heart. Further analyses at the single cell level (unpublished data) will help us to more accurately distinguish between these different cell types within the SAR.

Loss-of-function of three novel SAR-overexpressed genes - *pard6a*, *prom2*, and *atp1a1a.2,* affected both heart morphology and physiology in a dose-dependent manner. Although these candidate genes have not been previously implicated in cardiac development or function, evidences exist which support their specific involvement in this process. Notably, a mutant of aPKCλ, an interactor of Pard6a, is characterized by defects in heart tube assembly resulting from a disruption to adherens junction formation [44]. Therefore, it is reasonable to believe that a similar mechanism underlies the disrupted cardiac morphology observed in *pard6a* knockdown. Since Pard6a morphants develop bradycardia, it seems that Pard6a plays a role in the pacemaker function, but what this exact role is remains to be established. Interestingly, bradycardia was also observed as a result of *atp1a1a.2* knockdown despite its comparatively mild morphological phenotype. It is known that Na+/K + -ATPases are the major class of ion pumps in higher eukaryotes which is essential for maintaining proper heart rate by regulating intracellular ionic homeostasis of the heart cells [19, 45]. Interestingly, the ion gradient generated by Na+/K+ ATPase is also known to be essential for maintaining myocardial cell junction during heart morphogenesis [46], which may partly explain the cardiac phenotype observed in *atp1a1a.2* knockdown. It is worth noting that morpholino knockdown causes an overall reduction in gene expression, while different clinical genetic variants in human diseases could lead to a range of phenotypes. Therefore, higher resolution analyses of targeted mutagenesis on specific disease-causing variants are necessary to ascertain the mechanism of the candidate genes and create more accurate disease models.

## Conclusions

Thus, in this study, we characterized the molecular profile of the zebrafish SAR which exhibit pacemaker activity. The anatomical position of the SAR GFP-positive cells in transgenic line sqet33mi59BEt at the inflow region coincides with the previously reported SA pacemaker region in zebrafish heart. We show that the SAR overexpresses signature genes of mammalian SAN pacemaker, which include the *hcn4* ion channel which generates the pacemaker current, as well as other calcium and potassium gated channels known to be specific to pacemaker cells. Moreover, genes encoding components of the BMP and WNT signaling pathways, as well as members of the Tbx family which have previously been implicated in pacemaker development were also overexpressed in the SAR, which suggests conservation of molecular mechanism of pacemaker development and function in zebrafish and mammals. Finally, functional analyses of three SAR-overexpressed candidate genes suggested that they may play a role in heart development and physiology. Taken together, our data represents a valuable resource for the study of pacemaker function and heart diseases associated with the defect of this structure.

## Methods

### Zebrafish breeding

Wild-type and transgenic zebrafish sqet33mi59BEt were maintained in the zebrafish facility of the International Institute of Molecular and Cell Biology in Warsaw (License no.PL14656251 in the register of the District Veterinary Inspectorate in Warsaw), in line with the standard procedures and ethical practice recommended. Embryos were grown in embryo medium at 28 °C, staged according to standard morphological criteria [47]. Euthanasia of embryos was performed by overdosing with 200–300 mg/ml of tricaine (0.02% ethyl 3-aminobenzoate methanesulfonate) followed by freezing. All experimental procedures follow the standard protocols established by Polish Laboratory Animal Science Association. All experiments reported in this study involved zebrafish embryos younger than 120 h post-fertilization which do not fall into the regulatory frameworks of animal experimentation according to EU Directive 2010/63/EU on the protection of animals used for scientific purposes.

### Image acquisition using light-sheet fluorescence and confocal microscopes

Embryos at desired time point were anaesthetized using 0.16 mg/ml tricaine to stop heartbeat and then embedded into a ∼ 1 mm inner diameter glass capillary filled with 1.5% low-melting agarose (LMA) in embryo medium (0.03% Instant Ocean salt into double distilled water). Once agarose polymerized, the capillary was placed in the sample holder and inserted into a microscope chamber filled with embryo medium containing tricaine, and the embedded sample was pushed out of the capillary for imaging at a chamber temperature of 28 °C. Images were taken on ZEISS Light-sheet Z.1 with W Plan-Apochromat 20x/1.0. Z-stack (thickness 3.83 μm and exposure time 60 ms), saved in the LSM format, and then processed using ZEN software (Zeiss). For each z-stack, maximum intensity projections were generated. Confocal microscopy was performed on the LSM800 (Zeiss) by mounting embryos in 1.5% LMA in glass-bottom dishes.

### Embryonic heart isolation

Embryonic heart isolation was performed according to [48]. Three biological replicates of approximately 500 pooled 72 hpf embryos of sqet33mi59BEt transgenic line were prepared in a 5 ml low-binding tube and anaesthetized with tricaine (0.16 mg/ml in E3 medium). Washing was performed 5x in ice-cold E3 medium and 2x in ice-cold embryo destruction medium (EDM medium, L-15 medium from Gibco containing 10% FBS), followed by tissue dissociation by adding fresh EDM and pumping embryos repeatedly (25 times) through a 5 ml syringe with 19G needle. Media containing fragmented embryos as well as media obtained from rinsing syringe were filtered through a 100 μm nylon mesh (VWR), and flow-through was collected in a 30-mm glass petri dish. This flow-through was subsequently passed through a 40 μm nylon mesh to separate hearts from the debris and different fragments of bodies. The 40 μm mesh was inverted, and the retained material was washed off using EDM into a small petri dish. Intact and GFP positive hearts were collected using pipette under Olympus SZX16 fluorescence stereomicroscope to the low-binding tube with cold EDM medium and put on ice. The hearts were further processed using the following steps.

### Heart cell dissociation and fluorescence-activated cell sorting (FACS)

GFP labelled intact hearts were chemically dissociated into a single-cell suspension for FACS. Collected hearts were centrifuged at 1500 g for 10 min at 4 °C. The pellet was dissociated with 1 ml of the solution containing 0.05% trypsin, 0.2 mM EDTA and PBS, and further incubated in this solution for 6 min at room temperature. 1 ml of HBSS solution containing 10 mM Hepes and 2.5% (w/v) BSA was then added to stop the reaction, and the dissociated hearts were centrifuged at 1500 g for 10 min at 4 °C. Pellet was then resuspended in 150 μl FACS Max (Genlantis) and passed through 0.35 μm strainer. The dissociated cells were put on ice and sorted on the BD FACSAria II into GFP positive (GFP+) and GFP negative (GFP-) fractions directly into LS TRIZOL® (Invitrogen), three biological replicates each.

### RNA isolation from FACS sorted cells and assessment of RNA quantity and integrity

Total RNA was extracted from GFP+ and GFP− cells using TRIzol LS® (Invitrogen) according to the manufacturer’s protocol and followed by purification using RNA Clean & Concentrator (ZYMO Research). RNA yield and quality were assessed on the Agilent 2200 Tapestation with the High Sensitivity RNA Screen-Tape system according to the manufacturer’s protocol (Agilent Technologies). RNA Integrity Number (RIN^e^) was in the range of between 5.8 to 6.5 for all the samples used for RNA-seq. To obtain a high concentration, RNA was eluted in 7 μl of nuclease-free water.

### cDNA synthesis for library preparation and sequencing

cDNA synthesis was performed using SMART-Seq® v4 Ultra® Low Input RNA Kit for Sequencing (TakaRa) was used for cDNA synthesis. RNA-seq libraries were generated by Illumina Nextera XT kit according to manufacturer’s protocol and purified using Agencourt AMPure XP PCR purification beads (Beckman Coulter, USA). Fragment size distribution was assessed using High Sensitivity D1000 ScreenTape and Agilent 2200 TapeStation (Agilent Technologies, USA) and library was quantified with KAPA library quantification kit (Kapa Biosystems, USA). RNA-seq was performed with paired-end sequencing (2 × 75 bp reads) on the NextSeq 500 (Illumina, USA). The sequencing coverage was at least 10 million reads and 4 million reads for GFP+ and GFP-, respectively.

### Quantitative PCR

cDNA synthesis was performed using Roche SuperScript IV First-Strand Synthesis System (Thermo Fisher Scientific). Maximum amount of RNA (7 μl) was reverse transcribed to cDNA using random hexamers primers according to manufacturer’s instructions. The reaction was performed at 23 °C for 10 min, 55 °C for 10 min and 80 °C for 10 min. Relative mRNA expression was quantified by using FastStart SYBR Green master mix on the LightCycler 96 instrument (Roche Life Science) with specific primer sets.

### Data analysis

RNA-seq reads were mapped to the zebrafish genome assembly GRCz11 (Ensembl Genes 92). Gene expression (TPM values; transcripts per million) were quantified using Salmon [49] and pre-processed with quantile normalization and centralization [50]. Data were then used to train a self-organizing map (SOM), translating the expression profiles of all 19,854 genes measured into a two-dimensional map of 30 × 30 metagene profiles, where ‘profile’ denotes the vector of all samples’ expression values per gene or metagene. The SOM machine learning distributes the genes over the metagenes such that genes with similar profiles cluster together in the same or in closely located metagenes. After training, each metagene represents a cluster of single genes with very similar expression profiles, and the corresponding metagene profile can be interpreted as the mean profile averaged over all associated single gene profiles. The metagene expression values of each sample can be visualized by arranging them into a two-dimensional mosaic map representing the 30 × 30 metagenes, and by applying a color code presenting overexpressed metagenes in orange and red colors, intermediate ones in yellow and green, and underexpressed metagenes in light to dark blue. Each of those mosaic portraits exhibits characteristic spatial color patterns serving as a fingerprint of the transcriptional activity of the respective sample [51–53]. It is available as Bioconductor R-package ‘oposSOM’ [29]. We validated the oposSOM differential expression analysis using results of DESeq2 as an independent method analysis to compare between GFP+ and ROH fractions. Using a threshold of p_adj_ < 0.05 and − 1.5 > = log_2_FC > = 1.5, we found 858 genes upregulated and 1059 genes down-regulated in GFP+ compared to ROH (Table S6, see DESeq2_padj < 0.05). The results of DESeq2 and oposSOM largely agree with each other, with 63% (31 out of 49) of oposSOM SAR-enriched genes also identified as significantly upregulated in DESeq2 analysis.

### Intersection with GWAS dataset

To intersect SAR-overexpressed genes with GWAS dataset, SNP-phenotype Associations were downloaded from ClinVar (ftp://ftp.ncbi.nlm.nih.gov/pub/clinvar/vcf_GRCh38/archive_2.0/2020/). The database contains 1,319,815 Variant-Trait Associations. Zebrafish orthologs were identified for all known human SNP associated genes contained in ClinVar dataset using R BioMaRt Package. A total of 37,241 unique zebrafish orthologs were found, out of which 14,008 unique SNPs corresponded to SAR-overexpressed genes and 14,524 to SAR-underexpressed genes. Further, ClinVar data were intersected with CHD specific traits, resulting in 29 unique CHD specific SNPs from SAR-overexpressed and 7 from SAR-underexpressed gene lists.

### Morpholino analysis

Morpholino antisense oligomers (MO, Gene Tools, USA) were designed to block the translation of Pard6a (5′-GCGTTGTCCTGTGATTCCGTGACAT-3′), Prom2 (5′-AACTGCTTTCGTTCTGGACTTCATG-3′), and Atp1a1a.2 (5′-GTTCCAAGCCCCATTTTTCAAGATT-3′). For rescue experiment, candidate genes were cloned using a previously described method [54] using primers containing 6 sense mismatches at the morpholino recognition site: Pard6a (5′-TAATACGACTCACTATAAGGATAATCTAGAATGTCTCG CAACCATAGAACAACGCTGAAAAACGAG-3′ and 5′-GTTTAAACATTTAAATGGTACCTAGGATCCG ATTGTGCAACCCGTGTGATCC-3′ to amplify the coding sequence; 5′-GCAATTTTATCACGTTGTA GGCTTCACACTACATTTGGAG-3′ and 5′-GTTTAAACATTTAAATGGTACCTAGGATCCTGTACACCTT GACCCATCTG-3′ to amplify the 3′ untranslated region); Prom2 (5′-TAATACGACTCACTATAAG GATAATCTAGACCACAAAAATGAAATCTAGGACAAAGGCAGTTTCTTGGAG-3′ and 5′-GTTTAAAC ATTTAAATGGTACCTAGGATCCCGCTGCCTATCTTCTTCCATCTAGTTGATCAGACG-3′); Atp1a1a.2 (5′-TAATACGACTCACTATAAGGATAATCTAGAGAAATAAGACGTAAAAATGGGACTGGGAACAGG GAATG-3′ and 5′-GTTTAAACATTTAAATGGTACCTAGGATCCCCAGATTCTGTATCGCTTTTCTTTGA GAGTCTCTG-3′). Synthesis of mRNA was performed using mMessage mMachine kit with T7 RNA polymerase according to the manufacturer’s protocol (Thermo, USA). Two different concentrations of MO working solutions were prepared (2 ng/nl and 4 ng/nl) by diluting MO stock in deionized nuclease-free water (Polpharma, Poland) and adding 10% Phenol Red dye (Sigma Aldrich). One-cell stage embryos of ABxTL line were injected with 2 ng or 4 ng of gene-specific MO alone or together with 4 ng of p53 MO into the yolk and grown in standard conditions. For each MO type and concentration, experiment was performed with three replicates of at least 50 embryos each. MO rescue was performed by injecting embryos with a mixture of 4 ng MO and mRNAs (10 pg for *pard6a*, 50 pg for *prom2*, and 5 pg for *atp1a1a.2*). Mortality, phenotype and heartbeat were assayed at 24, 48, and 72 hpf in both groups. Embryos were mounted in 2% methylcellulose and imaged from lateral side in brightfield (SZX16, Olympus). For imaging of heart morphology, 72 hpf embryos were treated with 1x tricaine for 10 min to stop heartbeat and mounted individually in 2% methylcellulose. Bright field images from ventral side were taken under Imager.M2 microscope (Zeiss). Heartbeat rates were measured for 1 min under microscope by mounting each larvae in a drop of egg water on a petri dish. Experiment was performed at a stable temperature of 28 °C. Average heartbeat rate was obtained from 20 larvae for each experiment. Statistical significance between MO and control groups was determined using Student’s T-test.

### Whole mount in situ hybridization

Partial cDNA clones of *gfp, pard6a, prom2,* and *atp1a1a.2* were PCR amplified with the following primer pairs containing T7 promoter sequence overhang in the reverse primers: *gfp*: 5′- ATGGTGAGCAAGGGCGAGGAGC-3′ and 5′-CTTGTACAGCTCGTCCATGCCGA-3′; *pard6a*: 5′-CCTAGGTGGTCTGCGTCAAG-3′ and 5′-CCTAGGTAATACGACTCACTATAGGGGCATGTCCTCAC ACACTGGT-3′; *prom2*: 5′- TCCAACTGTGGTGGAGTGTTC-3′ and 5′- CCTAGGTAATACGACTCACT ATAGGGCTGCCTATCTTCTTCCATC-3′; *atp1a1a.2*: 5′- ACTTGTGCTTGCGTTTGTGG-3′ and 5′- CCTAGGTAATACGACTCACTATAGGGCGCAGCACAGCTCAATACAC-3′. *isl1a* was PCR amplified using the primer pair 5′-AGAGCCCATTTCGGCACGTC-3′ and 5′-TGTCGTTGGGTTGCTGCTGC-3′ and cloned into pGEMT-easy. Digoxygenin-labeled RNA antisense probes were synthesized using DIG RNA labeling mix (Roche, Switzerland) and T7 polymerase enzyme (Thermo, USA). Whole mount in situ hybridization was performed as previously described [55]. Images were captured on Leica M205FA (Leica, Germany) and Zeiss AxioImager.M2 (Zeiss, Germany).

## Supplementary Information

**Additional file 1: Figure S1.** FACS sorting and qPCR validation of GFP positive and GFP negative samples. (A) FACS sorting of the embryonic hearts at 72hpf shows distinct GFP positive and GFP negative cell populations. (B) qPCR validation of the samples was done to check for GFP and *neurogenin1* mRNA levels in the GFP positive and GFP negative samples. Y-axis represents fold change in percentage. qPCR was performed using ef-1α as endogenous control. RNA was extracted, reverse transcribed to cDNA and qRT-PCR reactions run using gene-specific primers to analyse mRNA levels.

**Additional file 2: Figure S2.** Comparison with mammalian SAN signatures. Heat map showing conserved mammalian SAN genes reported in [33] that have been compared with our SAR and ROH data set. 26 out of 39 genes among the conserved mammalian signature genes were also up-regulated in our dataset.

**Additional file 3: Figure S3.** Expression of candidate genes pard6a, prom2, and atp1a1a.2 and isl1a at 72hpf. Whole mount in situ hybridization revealed expression of *pard6a*, *prom2*, and *atp1a1a.2* in the heart, including the SAR at 72 hpf. Expression of *isl1a* was not detected in the heart at 72 hpf. Scale bars: 100 μm.

**Additional file 4: Figure S4.** Analysis of morpholino specificity. Specificity of MO against Pard6a, Prom2, and Atp1a1a.2 was assayed by co-injection with p53 MO and rescue experiment. Comparison between those injected with only 4 ng of gene-specific MO and those co-injected with p53 MO revealed no significant difference in morphological phenotype. Scale bar: 250 μm. Rescue experiment was performed by co-injecting 4 ng of MO with mRNA for each candidate gene (*pard6a* – 10 pg, *prom2* – 50 pg, *atp1a1a.2* – 5 pg). For all three candidate genes, an increase in proportion of normal or less severe phenotype was observed, suggesting rescue of the MO-induced phenotype. Scale bar: 50 μm.

**Additional file 5: Table S1.** List of genes under expressed in SAR or overexpressed in ROH.

**Additional file 6: Table S2.** List of differential GO sets, and their associated genes overexpressed among SAR (*p* < 0.1).

**Additional file 7: Table S3.** List of genes within the GO categories Calcium ion transport and potassium ion transport.

**Additional file 8: Table S4.** SAR-highly-enriched genes.

**Additional file 9: Table S5.** Comparison of SAR-overexpressed genes with mammalian SAN signatures.

**Additional file 10: Table S6.** Intersection between OposSOM and DESeq2 overexpressed genes (padj < 0.05).

**Additional file 11: Table S7.** List of SAN overexpressed genes associated with ClinVar phenotype.

## Data Availability

All sequencing data have been deposited in the GEO database under accession number GSE160398 (https://www.ncbi.nlm.nih.gov/geo/query/acc.cgi?acc=GSE160398). ClinVar data was obtained from ftp://ftp.ncbi.nlm.nih.gov/pub/clinvar/vcf_GRCh38/archive_2.0/2020/, the zebrafish genome reference GRCz11 is available from: https://www.ncbi.nlm.nih.gov/assembly/GCF_000002035.6/.

## Abbreviations

AVN: Atrioventricular node
BMP: Bone morphogenetic protein
CCS: Cardiac conduction system
CM: Cardiomyocyte
FACS: Fluorescence-activated cell sorting
FDR: False discovery rate
GFP: Green fluorescent protein
MO: Morpholino
ROH: Rest of heart
SAN: Sinoatrial node
SAR: Sinoatrial ring
TPM: Transcripts per million
