# Supplementary figures and images for: Transcriptome profile of the sinoatrial ring reveals conserved and novel genetic programs of the zebrafish pacemaker
Source: BMC Genomics. 2021 Oct 2;22:715. doi: 10.1186/s12864-021-08016-z (PMC8487553; doi:10.1186/s12864-021-08016-z)

A

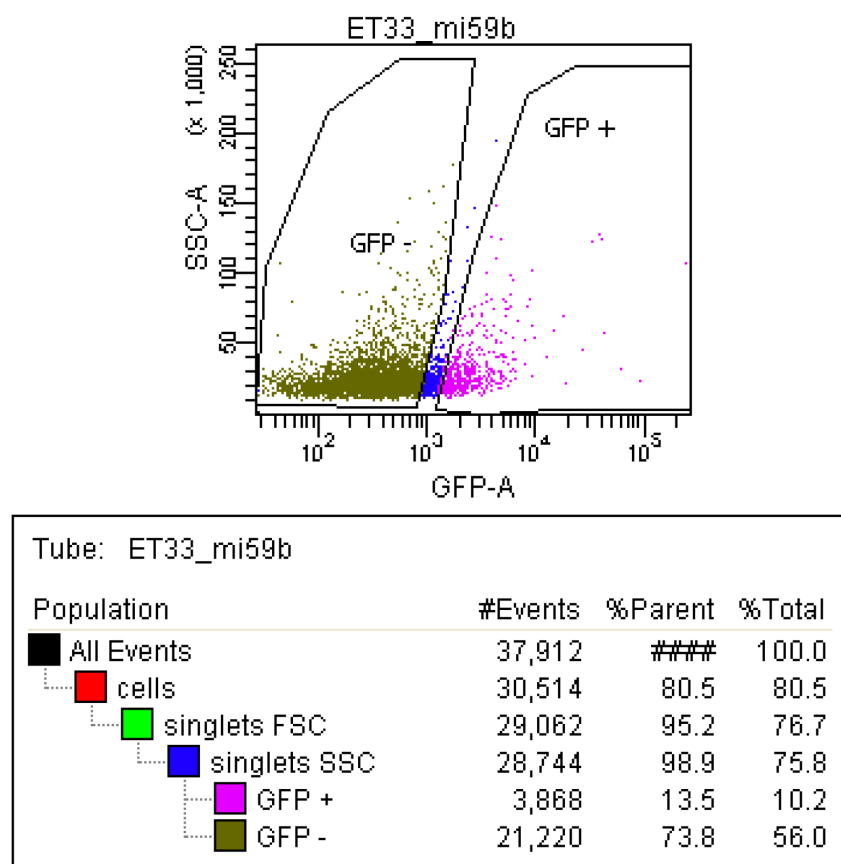

B

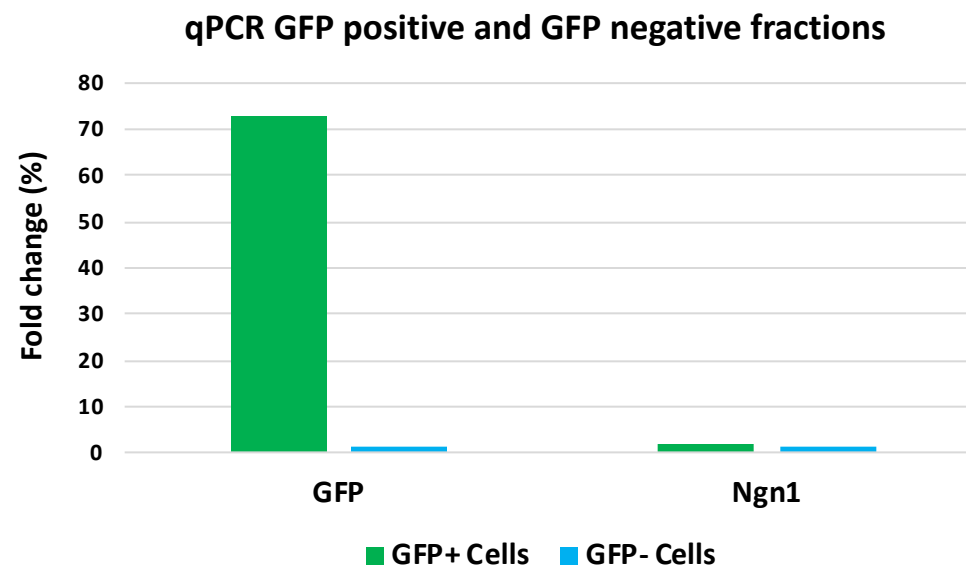

Supplement: Supplementary file 1 — Additional file 1: Figure S1. FACS sorting and qPCR validation of GFP positive and GFP negative samples. (A) FACS sorting of the embryonic hearts at 72hpf shows distinct GFP positive and GFP negative cell populations. (B) qPCR validation of the samples was done to check for GFP and neurogenin1 mRNA levels in the GFP positive and GFP negative samples. Y-axis represents fold change in percentage. qPCR was performed using ef-1α as endogenous control. RNA was extracted, reverse transcribed to cDNA and qRT-PCR reactions run using gene-specific primers to analyse mRNA levels. [file 12864_2021_8016_MOESM1_ESM.pdf]

# van Eif *et al.* SAN signatures

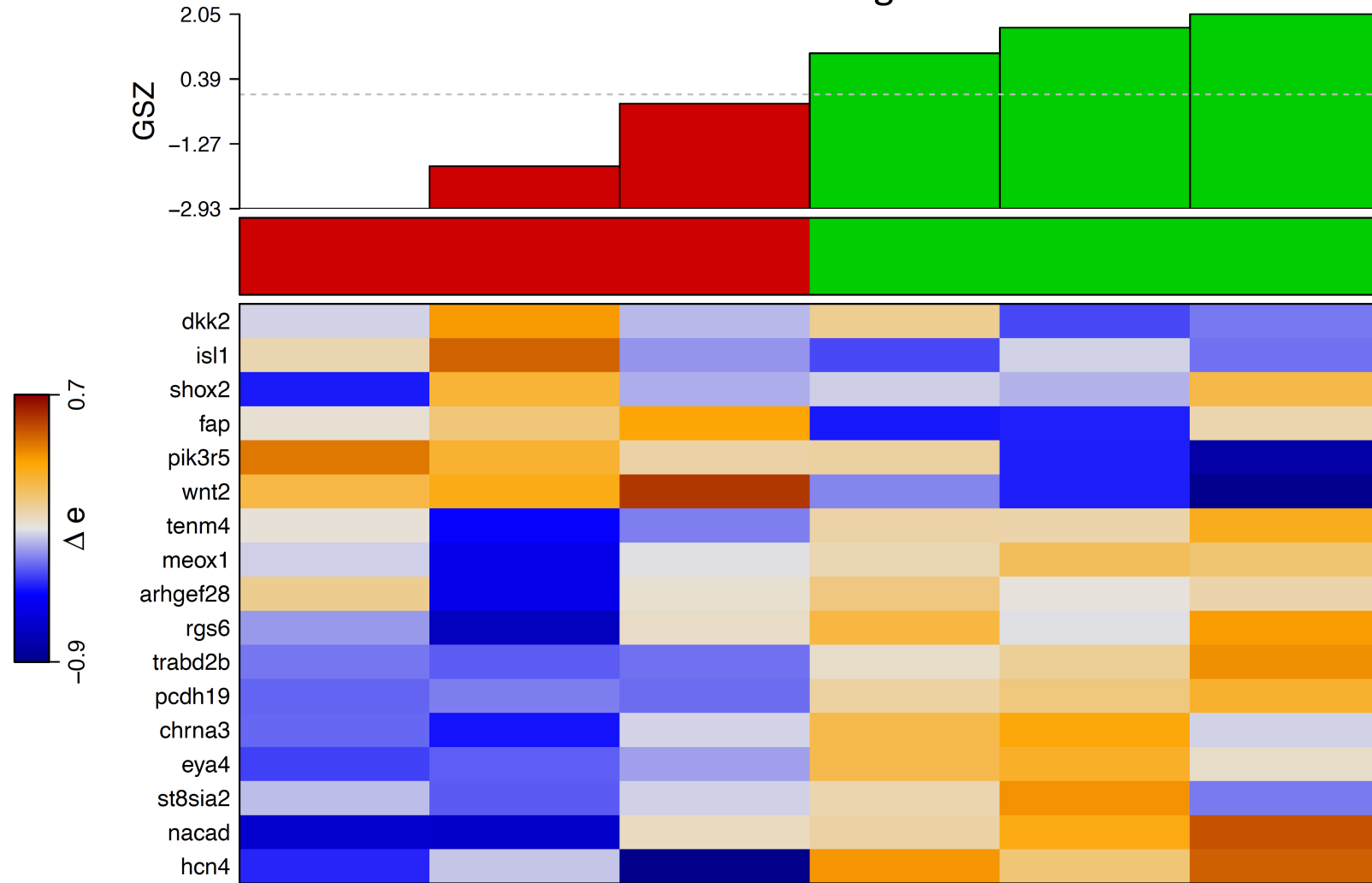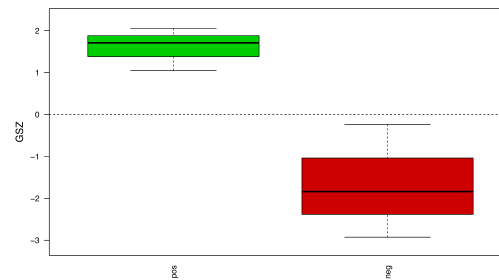

Supplement: Supplementary file 2 — Additional file 2: Figure S2. Comparison with mammalian SAN signatures. Heat map showing conserved mammalian SAN genes reported in [33] that have been compared with our SAR and ROH data set. 26 out of 39 genes among the conserved mammalian signature genes were also up-regulated in our dataset. [file 12864_2021_8016_MOESM2_ESM.pdf]

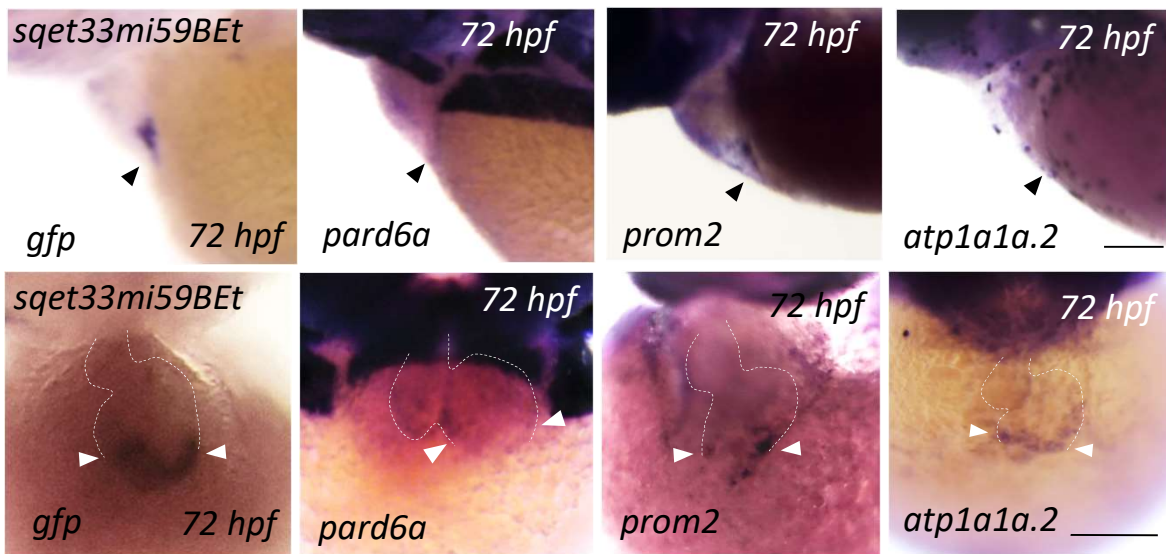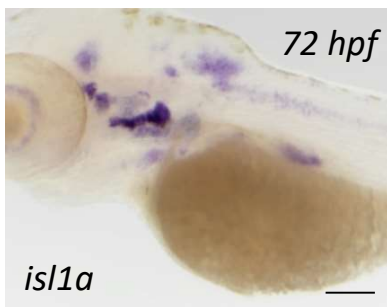

Supplement: Supplementary file 3 — Additional file 3: Figure S3. Expression of candidate genes pard6a, prom2, and atp1a1a.2 and isl1a at 72hpf. Whole mount in situ hybridization revealed expression of pard6a, prom2, and atp1a1a.2 in the heart, including the SAR at 72 hpf. Expression of isl1a was not detected in the heart at 72 hpf. Scale bars: 100 μm. [file 12864_2021_8016_MOESM3_ESM.pdf]
