# Supplementary material for: Transcriptome profile of the sinoatrial ring reveals conserved and novel genetic programs of the zebrafish pacemaker
Source: BMC Genomics. 2021 Oct 2;22:715. doi: 10.1186/s12864-021-08016-z (PMC8487553; doi:10.1186/s12864-021-08016-z)
Supplement: Supplementary file 4 — Additional file 4: Figure S4. Analysis of morpholino specificity. Specificity of MO against Pard6a, Prom2, and Atp1a1a.2 was assayed by co-injection with p53 MO and rescue experiment. Comparison between those injected with only 4 ng of gene-specific MO and those co-injected with p53 MO revealed no significant difference in morphological phenotype. Scale bar: 250 μm. Rescue experiment was performed by co-injecting 4 ng of MO with mRNA for each candidate gene (pard6a – 10 pg, prom2 – 50 pg, atp1a1a.2 – 5 pg). For all three candidate genes, an increase in proportion of normal or less severe phenotype was observed, suggesting rescue of the MO-induced phenotype. Scale bar: 50 μm. [file 12864_2021_8016_MOESM4_ESM.pdf]

Figure S4

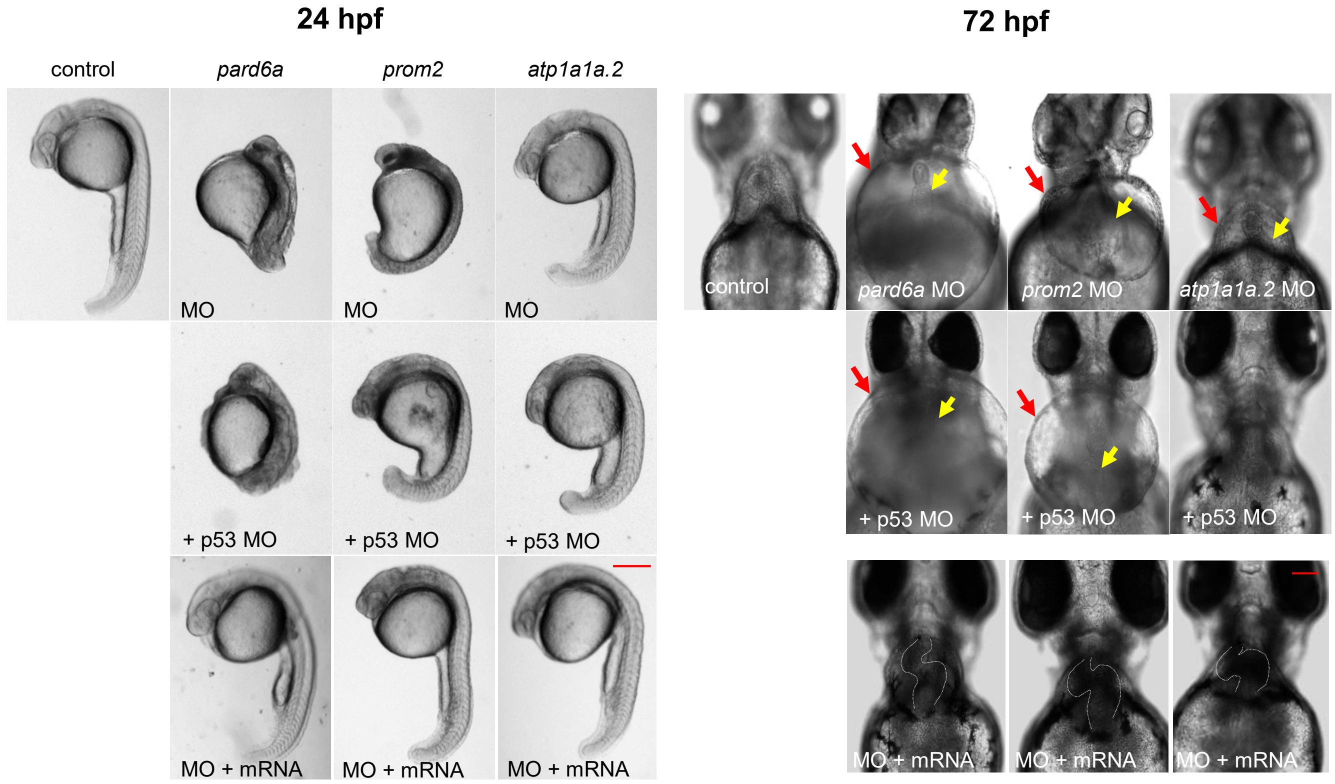

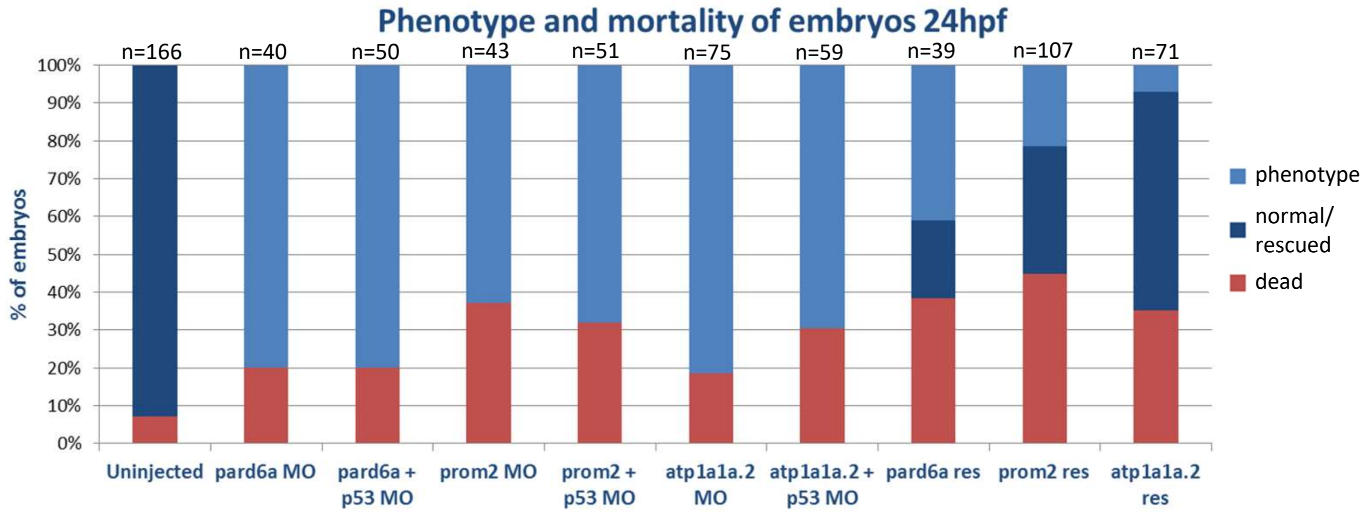

	Avg heartbeat (bpm)	Student's t-test
Uninjected	128.0819672	
Pard6a 4ng MO	57	1.59E-13
Pard6a 4ng MO +p53	100.6	1.93E-05
Atp1a1a.2 4ng MO	126.05	1.28E-02
Atp1a1a.2 4ng MO + p53	141.25	1.38E-07
Prom2 4ng MO	119.05	5.21E-01
Prom2 4ng MO + p53	123.25	8.20E-01
